# Nuclear genome sequencing reveals the highly intron-rich architecture of the chlorarachniophyte alga *Amorphochlora amoebiformis*

**DOI:** 10.1093/dnares/dsaf035

**Published:** 2025-11-28

**Authors:** Daichi Aoki, Honoka Saiki, Kenta Yamamoto, Shigekatsu Suzuki, Yoshihisa Hirakawa

**Affiliations:** Graduate School of Life and Environmental Sciences, University of Tsukuba, 1-1-1 Tennodai, Tsukuba 305-8572, Japan; Graduate School of Life and Environmental Sciences, University of Tsukuba, 1-1-1 Tennodai, Tsukuba 305-8572, Japan; Graduate School of Life and Environmental Sciences, University of Tsukuba, 1-1-1 Tennodai, Tsukuba 305-8572, Japan; Biodiversity Division, National Institute for Environmental Studies, 16-2 Onogawa, Tsukuba 305-8506, Japan; Institute of Life and Environmental Sciences, University of Tsukuba, 1-1-1 Tennodai, Tsukuba 305-8572, Japan; Institute of Life and Environmental Sciences, University of Tsukuba, 1-1-1 Tennodai, Tsukuba 305-8572, Japan

**Keywords:** microalgae, plastid, nucleomorph, endosymbiosis, gene transfer

## Abstract

Chlorarachniophyte algae possess complex plastids derived from endosymbiosis between a cercozoan protist and green alga. As evidence of this event, remnant nucleus of the endosymbiont, nucleomorph, is present in the plastid intermembrane space. Chlorarachniophytes are excellent models to study genome evolution via endosymbiosis. Although the three organelle genomes of mitochondrion, plastid, and nucleomorph have been sequenced in several chlorarachniophyte species, nuclear genome information is currently limited to *Bigelowiella natans*. To gain insights into the genome diversity and evolution of chlorarachniophytes, we sequenced the nuclear genome of another chlorarachniophyte, *Amorphochlora amoebiformis*. Its size is approximately 214 Mb, which is more than twice that of *B. natans*. Remarkably, three-quarters of the nuclear genome encodes spliceosomal introns, indicating its highly intron-rich structure compared to other known eukaryotic genomes. Single nucleotide polymorphism analysis revealed that *A. amoebiformis* possessed a diploid nuclear genome, unlike the haploid genome of *B. natans*. Additionally, we identified organellar DNA fragments within the nuclear genome, suggesting recent DNA migration from the three organelles to the nucleus. Overall, our findings reveal that chlorarachniophyte nuclear genomes differ substantially in size, structure, and ploidy across species, and provide evidence of ongoing endosymbiotic gene transfer.

## 1. Introduction

Primary endosymbiosis between a heterotrophic protist and photosynthetic cyanobacterium gave rise to a primitive plastid-bearing alga,^1^ which eventually evolved into Archaeplastida encompassing chlorophytes, rhodophytes, glaucophytes, and streptophytes.^2^ Subsequently, various algal lineages with complex plastids evolved via multiple secondary endosymbioses, in which eukaryotic hosts integrated a eukaryotic alga of chlorophytes and rhodophytes.^3,4^ These endosymbiotic events resulted in the phylogenetic diversity of eukaryotic algae.^5^ Chlorarachniophytes are marine unicellular algae that possess complex plastids originated by secondary endosymbiosis between a cercozoan protist of the Rhizaria supergroup and a green alga closely related to Bryopsidales.^6,7^ The most prominent feature of chlorarachniophytes is a remnant of the endosymbiont’s nucleus in the plastid intermembrane space.^8^ This organelle, the so-called nucleomorph, contains a highly reduced eukaryotic genome encoding several hundred genes.^9^ Notably, chlorarachniophytes possess four distinct genomes (plastid, mitochondrial, nuclear, and nucleomorph genomes) in a single cell. This unique feature makes them attractive models to study genome evolution via endosymbiosis.

To date, nine genera and 15 species of chlorarachniophytes have been described, all of which are marine unicellular organisms.^10,11^ They inhabit diverse marine environments, ranging from coastal to open ocean regions, and from tropical to temperate zones. Cell morphology varies by species, and three vegetative cell types (flagellate, amoeboid, and coccoid) have been identified.^10^  *Bigelowiella natans* cells are swimming with a single flagellum,^12^ whereas *Gymnochlora stellata* exhibits benthic amoeboid cells.^13^ Some species exhibit multiple cell types during their life cycle. For instance, *Lotharella globosa* typically exists as walled coccoid cells, but occasionally transforms into flagellate cells in culture.^14^

Chlorarachniophytes possess four evolutionarily distinct genomes, and organelle genomes have been sequenced in multiple species, with plastid, nucleomorph, and mitochondrial genomes sequenced in five, four, and three species, respectively^6,15–20^ Additionally, transcriptomic data of nine species are available, thanks to the Marine Microbial Eukaryote Transcriptome Sequencing Project.^21^ However, to date, nuclear genome sequence has been reported only in *B. natans*.^22^ Its nuclear genome is approximately 95 Mb in size and carries over 21,000 protein-coding genes rich in spliceosomal introns (average 7.8 introns/gene); alternative splicing events have been reported.^22^ Thousands of organelle-targeted proteins are encoded by nuclear genes, many of which have been derived from endosymbiont genomes via endosymbiotic gene transfer (EGT). Notably, the presence of nuclear mitochondrial DNA fragments (NUMTs) suggests ongoing mitochondrial DNA transfer to the nucleus in this species.^22^ However, nuclear copies of plastid DNA (NUPTs) and nucleomorph DNA (NUNMs) were not detected in *B. natans*.

*Amorphochlora amoebiformis* (formerly *Lotharella amoebiformis*)^23^ is an amoeboid cell with multiple plastids,^24^ unlike flagellate *B. natans* containing a single plastid. *A. amoebiformis* is an experimental model of chlorarachniophytes due to the availability of transient genetic transformation.^25,26^ To date, organelle genomes have been sequenced in *A. amoebiformis*.^18,20^ Although nuclear genome sequencing of this species was attempted using Illumina short reads, the resulting de novo assembly was highly fragmented, producing over 60,000 scaffolds with an N50 of 8 kbp.^27^ In this study, to gain further insights into the nuclear genome diversity and evolution of chlorarachniophytes, we performed long-read sequencing using the Oxford Nanopore technology and successfully assembled the nuclear genome of *A. amoebiformis*, revealing its highly intron-rich architecture.

## 2. Materials and methods

### 2.1. Cultivation

*Amorphochlora amoebiformis* (CCMP2058 strain) was grown in Erlenmeyer flasks with 200 mL ESM medium^28^ under RGB-LED illumination (60 to 80 µmol photons m^−2^ s^−1^) at 22 °C on a 12-h light/12-h dark cycle. The cells were subcultured every 2 to 3 wks.

### 2.2. DNA extraction and sequencing

*Amorphochlora amoebiformis* cells were collected from a 6-d-old culture by brief centrifugation. Total 200 mg of cell pellet was resuspended in 5 mL of lysis buffer (50 mM Tris-HCl, 100 mM EDTA, 200 mM NaCl, 1% Sarcosyl, 0.2 mg/mL protease K, pH 8.0) and incubated at 55 °C for 1 h. Nucleic acids were extracted using phenol:chloroform (1:1), followed by 100% chloroform. DNA was purified by CsCl density gradient ultracentrifugation, as previously described.^29^ Briefly, 4.25 g CsCl was dissolved in 3.7 mL of the aqueous phase containing nucleic acids, followed by the addition of 10 µL SYBR Safe DNA Gel stain (Thermo Fisher Scientific, Waltham, MA, USA). The solution was subjected to ultracentrifugation at 50,000 rpm for 22 h using the VTi65-2 rotor (Beckman Coulter, Brea, CA, USA), and DNA was visualized under blue LED light and collected from a single band (Fig. S1). DNA was recovered by ethanol precipitation and resuspended in distilled water at a final concentration of 1 µg/µL. DNA concentration was determined using a Qubit dsDNA BR Assay Kit on a Qubit 3.0 fluorometer (Thermo Fisher Scientific).

Nanopore long-read sequencing was performed using a MinION Mk1C device with MinKNOW software v22.10.7 (Oxford Nanopore Technologies, Oxford, UK). Library preparation and sequencing were conducted according to the manufacturer’s standard protocols. Four sets of Flongle Flow Cell R9.4.1 (Oxford Nanopore Technologies) were used for preliminary sequencing. Purified DNA was partially sheared via 20 passes through a 26G needle, and then short DNA fragments were removed using a Short Read Eliminator Kit (PacBio, Menlo Park, CA, USA) as previously described.^30^ A sequencing library was prepared using a Ligation Sequencing Kit SQL-LSK109 (Oxford Nanopore Technologies). Additional sequencing was performed using a MinION Flow Cell R10.4.1 (Oxford Nanopore Technologies) with non-sheared intact DNA and a Ligation Sequencing Kit V14 SQK-LSK114 (Oxford Nanopore Technologies). Basecalling was performed using Guppy software v6.4.6 (Oxford Nanopore Technologies) with the super accuracy mode (minimum Q-score of 10). Raw sequence data were deposited in the DDBJ Sequence Read Archive (accession numbers: DRR687620 and DRR687621).

### 2.3. RNA extraction and sequencing

Actively growing cells were collected from 2-d-old cultures every 6 h (at 0 and 6 h in the dark and light phases). Total RNA was extracted from these cells using TRIzol reagent (Thermo Fisher Scientific), according to the manufacturer’s instruction. Library preparation and sequencing were performed by Rhelixa, Inc. (Tokyo, Japan). Briefly, mRNA enrichment was performed using an NEBNext Poly(A) mRNA Magnetic Isolation Module (New England Biolabs, Ipswich, MA, USA), and a sequencing library was constructed using an NEBNext Ultra II Directional RNA Library Prep Kit for Illumina (New England Biolabs). Paired-end short reads were obtained using an Illumina sequencing platform (NovaSeq 6000). Raw RNA sequencing (RNA-seq) data were deposited in the DDBJ Sequence Read Archive (accession numbers: DRR687622–DRR687625).

### 2.4. Genome assembly and annotation

In total, 5.2 Gb nanopore reads (approximately 25-fold coverage) were assembled using Raven v1.8.1.^31^ Long-read assemblies were polished with nanopore reads using Medaka v1.7.2 (Oxford Nanopore Technologies). The resulting consensus was further polished with Illumina short reads (GenBank accession numbers: SRR14100013) using Nextpolish v1.4.1.^32^ The Illumina reads were trimmed using fastp v0.23.4.^33^ Contigs of organellar genomes were manually removed from the assembly based on sequence identity to the plastid DNA (GenBank accession number: LC781622) and nucleomorph DNAs (AB996602, AB996603, and AB996604); no contig corresponding to the mitochondrial DNA (LC781623) was detected in the assembly. Potential bacterial contamination was assessed using Whokaryote version 1.1.2,^34^ however, no bacterial sequences were detected. Repeat elements of the assembly were annotated using RepeatModeler version 2.0.2^35^ and subsequently masked using RepeatMasker version 4.1.2 (https://www.repeatmasker.org/) with default settings. The resulting assembly was annotated using funannotate pipeline version 1.8.11 (https://github.com/nextgenusfs/funannotate). Before annotation, we run the funannotate train with the option –max_intronlen 200000, using RNA-seq reads trimmed with fastp version 0.23.2.^33^ In this step, Trinity version 2.8.5^36^ and PASA version 2.5.3^37^ were used to generate transcript assembly. The completeness of the genome assembly was assessed using Benchmarking Universal Single-Copy Orthologs (BUSCO) v6.0.0 with the eukaryota_odb12 dataset.^38^ Both the assembled contigs and annotated proteins were used for this analysis. Additionally, BUSCO scores of the *B. natans* genome were estimated using the nuclear DNA (nDNA) contigs (GenBank accession number: GCA_000320545.1) and protein sequences (Dryad: https://doi.org/10.5061/dryad.6n332).

### 2.5. Gene expression analysis

Illumina RNA-seq reads of *A. amoebiformis* at each time point (dark: 0 and 6 h; light 0 and 6 h) were trimmed using fastp v0.23.4,^33^ and mapped to the nuclear genome contigs using HISAT2 v2.2.1.^39^ Transcript abundance of each gene at each time point was quantified using StringTie v2.2.1^40^ as transcripts per million (TPM) values. TPM values of *B. natans* nuclear genes were calculated using our previous RNA-seq data.^41^

### 2.6. Single nucleotide polymorphism analysis

Single nucleotide polymorphisms (SNPs) were identified as previously described.^42^ Briefly, repeat sequences in the nuclear contigs were annotated and masked using RepeatModeler v2.0.3^35^ and RepeatMasker v4.1.2 with the -nolow option. Illumina short reads were mapped to the nuclear genome using minimap2 v2.17 (-ax sr –secondary = no).^43^ SNPs were detected using VarScan2 v2.4.4^44^ with the following parameters: –min-coverage 20 –min-var-freq 0.01 –min-freq-for-hom 0.99. Allele frequencies at each SNP position were extracted from the VCF files, and histograms were plotted using RStudio v2024.09.0 (Posit Software, MA, USA).

### 2.7. K-mer frequency analysis

Using total 26.1 Gb Illumina short reads (GenBank accession numbers: SRR14100013, DRR796689, and DRR796690) of *A. amoebiformis* and 8.5 Gb Illumina short reads (SRR14100056) of *B. natans*, k-mers were counted using jellyfish v2.3.1^45^ at *k* = 21. The Illumina reads were trimmed using fastp v1.0.1.^33^ K-mer spectra were visualized using GenomeScope2.^46^

### 2.8. Phylogenetic analysis

To construct phylogenetic trees for mitochondrial NAD9 and RPS11 proteins, homologous sequences were obtained from the NCBI database. Sequence alignments were generated using the L-INS-i method in the MAFFT package,^47^ and poorly aligned positions were removed using trimAl with the automated1 option.^48^ Phylogenetic analyses were performed using IQ-TREE v2.3.6 with the MFP option.^49^ Branch support values were evaluated with 100 standard non-parametric bootstrap replicates. Maximum likelihood trees were visualized using FigTree v1.4.4.

### 2.9. Subcellular localization of fluorescent-tagged protein

Plasmids were constructed to express fusion proteins of a nucleus-encoded mitochondrial protein and yellow fluorescent protein (YFP) or cyan fluorescent protein (CFP). Total RNA was extracted from *A. amoebiformis* cells, as described above, and cDNA was synthesized using Superscript IV reverse transcriptase (Thermo Fisher Scientific) with oligo (dT)_20_ primers. Then, cDNA fragments of mitochondrial *nad9* (AAMOR_001647-T1) and *rps11* (AAMOR_015054-T1) were amplified by PCR using KOD One PCR Master Mix (TOYOBO, Osaka, Japan). Each fragment was inserted upstream of the YFP-coding sequence in the pAaTu-YFP vector^20^ using In-Fusion Snap Assembly Master Mix (Takara Bio, Shiga, Japan). The inserted sequences were verified by Sanger sequencing. Expression cassettes for YFP fusion proteins were subcloned into another plasmid expressing mitochondria-targeted CFP, HisRS77124-2ndMet + CFP.^50^ All plasmids were cloned into *Escherichia coli* DH5α cells (TOYOBO)

Transfection-grade plasmid DNA was purified from *E. coli* cells using a ZymoPURE II Plasmid Midiprep Kit (Zymo Research, Irvine, CA, USA), and *A. amoebiformis* cells were transfected as previously described.^26^ Briefly, approximately 1 × 10^7^ cells were resuspended in 100 µL of Gene Pulser Electroporation Buffer (Bio-Rad, Hercules, CA, USA) with 15 mg sucrose, followed by the addition of 10 µL plasmid DNA (5 µg/µL). The mixture was pulsed at 120 V for 25 ms in a 0.2 cm cuvette using a Gene Pulser Xcell electroporation system (Bio-Rad). The cells were immediately transferred to glass-bottomed dishes (AGC Techno Glass, Shizuoka, Japan) containing fresh ESM medium and incubated under the culture conditions described above. After 24 h incubation, YFP-expressing cells were observed under an Olympus IX71 inverted fluorescence microscope (EVIDENT, Nagano, Japan). Confocal images were obtained using an inverted Zeiss LSM 510 laser scanning microscope (Carl Zeiss, Jena, Germany) and analyzed using ZEN 2012 software (Carl Zeiss).

## 3. Results and discussion

### 3.1. *A. amoebiformis* nuclear genome sequencing

Nanopore long-read sequencing was performed using the total DNA extracted from the chlorarachniophyte, *A. amoebiformis* (CCMP2058 strain), and nDNA sequences were assembled into 567 contigs with an N50 of 693 kb and a total length of 214.2 Mb (Table 1). The largest contig was 2.6 Mb in length and contained telomeric repeats of [5′-TAACCC-3′] at both ends. BUSCO scores were estimated to be 58.1% and 92.2% based on the assembled DNA and annotated protein sequences, respectively (Table 1; Table S1), indicating the high completeness of the assembly.

**Table 1.** Nuclear genome composition of the two chlorarachniophytes, *A. amoebiformis* and *B. natans*.

	*A. amoebiformis* (this study)	*B. natans* (Curtis et al.)^22^
Assembly size (Mbp)	214.2	91.4 (94.7)^a^
Sequencing method	Nanopore & Illumina	Sanger & 454
Number of contigs	567	3,736
N50 length (kbp)	693.0	59.5
GC content (%)	27	45
Protein-coding genes	17,505	21,708
Complete BUSCOs (proteins)	92.2%	86.0%
Complete BUSCOs (genome)	58.1%	60.5%
Average gene length (bp)	10,757	2,834
Exons		
Average number per gene	17.5	8.8
Average length (bp)	101	159
Total length (Mbp)	30.8	30.5
Introns		
Average number per gene	16.5	7.8
Average length (bp)	546	184
Total length (Mbp)	158.3	31.3
Intergenic regions		
Total length (Mbp)	25.1	32.9

The genome information of *Bigelowiella natans* is according to the previous study by Curtis et al.^22^ The BUSCO scores of *B. natans* were obtained by BUSCO v. 6.0.0 using the eukaryota_odb12 dataset in this study.

^a^The number in parentheses represents the total size of assembled scaffolds.

**Fig. 1.**
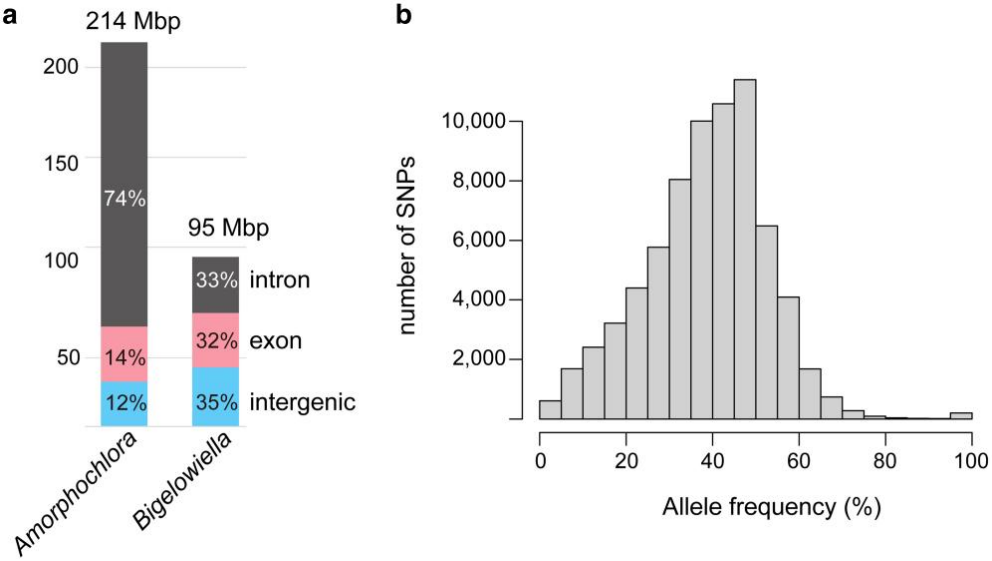
Intron-rich architecture of the *A. amoebiformis* nuclear genome. a) Comparison of coding and non-coding regions between nuclear genomes of *A. amoebiformis* and *B. natans.* b) Histogram showing allele frequency at 717,620 polymorphic sites. The *x*-axis indicates allele frequency, and the *y*-axis represents the number of SNPs.

The nuclear genome of *A. amoebiformis* was predicted to contain 17,505 protein-coding genes, approximately 14% of which generate multiple transcript variants, as well as 105 tRNA genes. In another chlorarachniophyte, *B. natans*, 21,708 protein-coding genes were predicted in a nuclear genome of 91.4 Mb.^22^ Interestingly, nuclear genome size of *A. amoebiformis* was more than twice that of *B. natans* despite the presence of fewer protein-coding genes. This size difference was due to the presence of massive spliceosomal introns in *A. amoebiformis* (Table 1; Fig. 1a). *B. natans* harbors a certain number of spliceosomal introns with an average of 7.8 introns/gene and a total intron size of 31 Mb. Comparatively, *A. amoebiformis* possessed a higher number of introns, average 16.5 introns/gene, with a total intron size of 158 Mb (Table 1). Furthermore, average intron length in *A. amoebiformis* was approximately three times greater than that in *B. natans* (Table 1). Notably, introns accounted for 74% of the *A. amoebiformis* nuclear genome, making it the most intron-rich eukaryotic genome identified to date. Even in the intron-rich genomes of dinoflagellates, *Polarella glacialis*^51^ and *Breviolum minutum*,^52^ introns account for up to 60% of the nuclear genome. A conspicuous feature of the *A. amoebiformis* genome is the widespread presence of simple sequence repeats (SSRs), such as [AT]_n_, which are largely absent in the *B. natans* genome (Fig. S2). These SSRs likely interfered with de novo assembly using Illumina short reads in a previous study by Nelson et al.^27^

### 3.2. Diploid genome of *A. amoebiformis*

The nuclear genome of *A. amoebiformis* exhibits characteristics of diploidy, unlike that of *B. natans*, which is haploid.^22^ When Illumina short reads (GenBank accession number: SRR14100013)^27^ were aligned to the nuclear contigs of *A. amoebiformis*, 717,620 SNPs were detected, corresponding to approximately 3 variants per 10 kbp. Genome-wide allele frequencies of the SNPs exhibited a mountain-shaped distribution, with a peak around 50% (Fig. 1b). Allele frequency histograms for individual contigs also showed peaks around 45 to 50% (Fig. S3). Considering that the *A. amoebiformis* strain was established as a clonal culture from a single amoeboid cell and the observed SNP allelic ratios were close to 1:1, *A. amoebiformis* possibly possessed a diploid nuclear genome. Moreover, we performed k-mer frequency analysis using Illumina short reads. A bimodal distribution of k-mer counts was observed in *A. amoebiformis* (Fig. S4), supporting the diploid hypothesis.

Sexual reproduction has been documented in two chlorarachniophytes, *Chlorarachnion reptans*^53^ and *Cryptochlora perforans*,^54^ with haploid zoospores developing from diploid coccoid cells in *C. perforans*. Sexual reproduction is not known in *A. amoebiformis*, however, flagellated cells have been observed very rarely when cells are subcultured onto new medium.^24^ This implies that the flagellated cells may represent a haploid stage developing from diploid amoeboid cells in *A. amoebiformis*.

### 3.3. Diurnal transcriptomes of *A. amoebiformis*

Nuclear gene expression was assessed using RNA-seq data. Total RNA was extracted from the actively growing *A. amoebiformis* cells maintained under a 12:12-h light:dark cycle, with samples collected every 6 h for 24 h (at 0 and 6 h in the light and dark phases). RNA-seq was performed, and Illumina short reads were mapped to the nuclear genome. TPM values were calculated for each gene at each time point (Table S2). TPM values varied from 0 to >10,000 across the nuclear genes, with an average of approximately 56. Over 800 genes, including many hypothetical protein-coding genes, exhibited extremely low TPM values (< 0.1). Highly expressed genes (TPM > 2,000) were primarily annotated as photosynthesis-related proteins (eg, light-harvesting complex proteins and carbon fixation enzymes) and housekeeping proteins (eg, tubulin and translation factor proteins). TPM values of these photosynthesis-related genes exhibited diurnal fluctuations during the light/dark cycle, consistent with a previous report on *B. natans* (Table S3).^41^

### 3.4. Extensive intron gains in *A. amoebiformis*

**Fig. 2.**
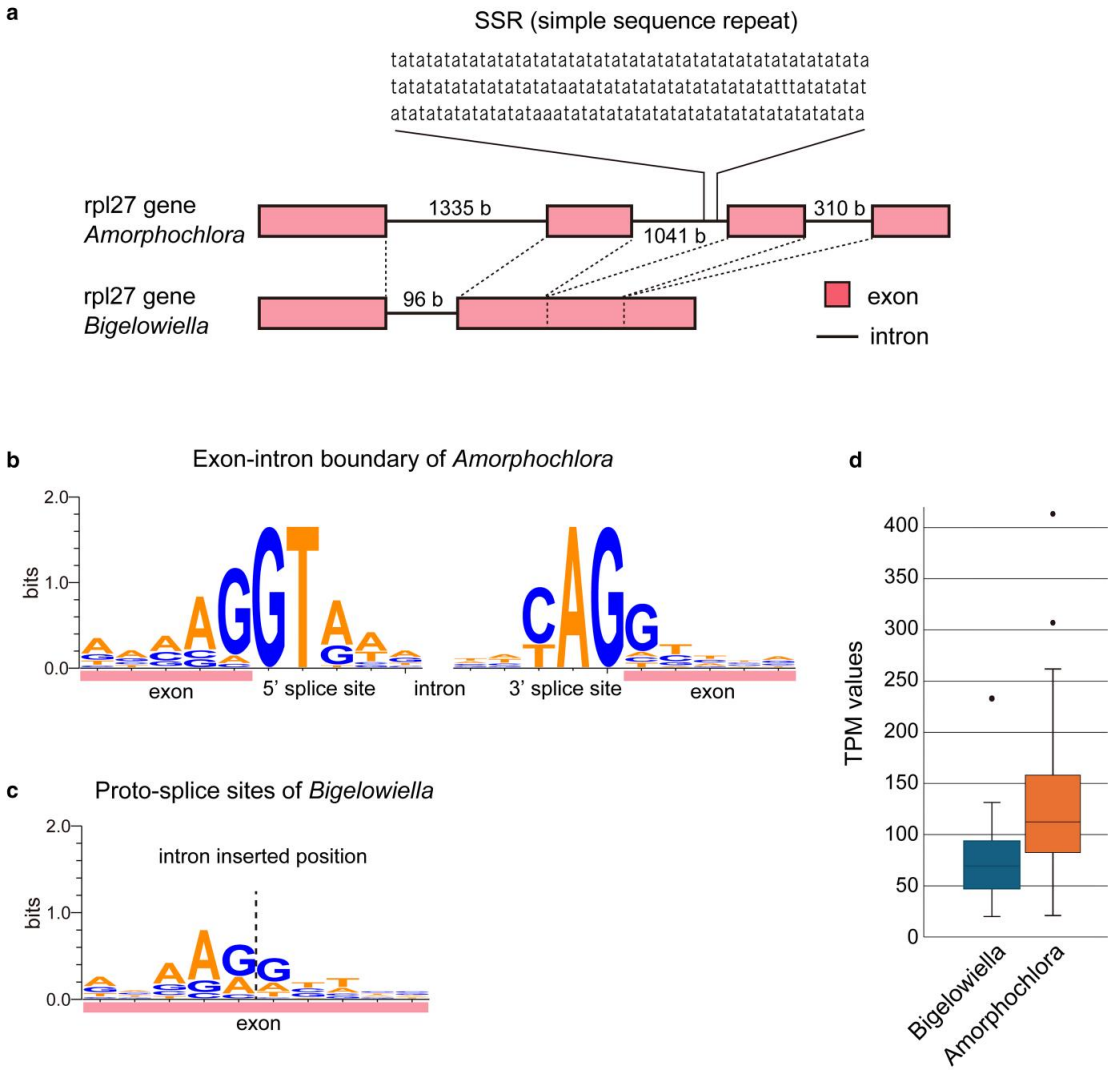
Gain of spliceosomal introns in ribosomal protein genes of *A. amoebiformis*. a) Comparison of intron sizes and positions in *rpl27* genes between *A. amoebiformis* and *B. natans*. b) Conserved motif at exon-intron boundaries of 26 inserted introns in ribosomal protein genes of *A. amoebiformis*. c) Putative proto-splice sites in ribosomal protein genes of *B. natans* corresponding to the 26 inserted introns of *A. amoebiformis*. Sequence logos were obtained by WebLogo.^55^ d) TPM values of 35 spliceosome protein genes in each chlorarachniophyte. Expression levels differ significantly between the two species (*P* < 0.01 by paired *t*-test).

To investigate the evolution of abundant introns in the *A. amoebiformis* nuclear genome, we compared the intron positions and lengths of conserved ribosomal protein genes between the two chlorarachniophyte species, *B. natans* and *A. amoebiformis*. A total of 26 introns in 12 ribosomal protein genes were found exclusively in *A. amoebiformis* (Table S4). These introns carried canonical GT and AG boundary motifs (Fig. 2b), with SSRs of [AT]_n_ (*n* > 10) observed in half of them (Fig. 2a). Sequence alignments revealed that nine of these introns were inserted at sites corresponding to the AG|GN motif in *B. natans* genes (Fig. 2c). These findings suggest that lineage-specific intron gains occurred in *A. amoebiformis*, possibly via proto-splice sites resembling the exon-intron boundary (AG|GT..intron..AG|G), thereby minimizing coding sequence disruption. To explore the possible relationship between intron abundance and splicing capacity, we compared the expression levels of splicing-related genes between the two species. The average TPM value of 35 spliceosome proteins was higher in *A. amoebiformis* than in *B. natans* (Fig. 2d;  Table S5), consistent with the increased number of introns observed in *A. amoebiformis*.

Next, we compared the lengths of 12 conserved introns at identical positions in the ribosomal protein genes of both species. The average intron size in *A. amoebiformis* was approximately 340 bp, about twice that in *B. natans* (∼160 bp). Collectively, these data suggest that an expansion in both the intron size and number contributes to the highly intron-rich genome structure of *A. amoebiformis*. However, the possibility of a common chlorarachniophyte ancestor possessing an intron-rich genome, with *B. natans* subsequently undergoing extensive intron loss, cannot be excluded. Further genome sequencing of other chlorarachniophyte species is necessary to clarify the evolutionary trajectory of introns in this lineage.

It is believed that intron-generating transposable elements, termed “Introners”, are responsible for rapid and extensive intron gain in diverse eukaryotes.^56^ Introner-derived spliceosomal introns often possess short terminal inverted repeats.^57,58^ Lineage-specific introns in the ribosomal protein genes of *A. amoebiformis* probably do not originate from Introners, because of the absence of conserved sequences and terminal inverted repeats. However, it is noteworthy that the *A. amoebiformis* genome contains an elevated number of genes associated with DNA transposition. Orthologous gene identification using OrthoFinder^59^ revealed that *A. amoebiformis* possessed 34 orthologs of DNA transposase belonging to the DDE superfamily compared to only eight genes found in *B. natans* (Fig. S5). Moreover, abundant interspersed repeats including DNA transposons were identified in the *A. amoebiformis* genome using RepeatModeler and RepeatMasker (Fig. S2). However, it remains unclear whether DNA transposons contribute to intron gains in *A. amoebiformis*.

### 3.5. Conserved flagellar protein genes in *A. amoebiformis*

**Fig. 3.**
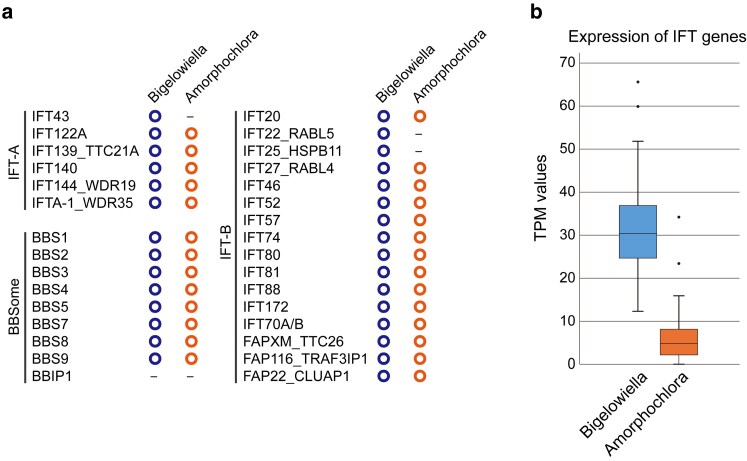
Genes for IFT complexes in two chlorarachniophytes. a) A total of 30 and 27 IFT genes were detected in *B. natans* and *A. amoebiformis*, respectively. Colored circles indicate the presence of each gene in the genomes. b) Box plots showing expression levels of IFT genes in *B. natans* and *A. amoebiformis*. The *y*-axis indicates TPM values. Boxes span the first and third quartiles, center lines indicate the medians, whiskers represent the full data range excluding outliers (dots). Expression levels differ significantly between the two species (*P* < 0.01 by paired *t*-test).

Flagellated cells have been reported as rare morphotypes in *A. amoebiformis*,^24^ however, we have never observed such cells in the past two decades (the stain was subcultured every few weeks). To assess whether *A. amoebiformis* retains the potential to generate swimming cells, we searched its nuclear genome for genes encoding flagellum-specific proteins. The intraflagellar transport (IFT) complex is an essential component of eukaryotic flagella/cilia, with 31 conserved IFT proteins identified across diverse eukaryotes.^60^ Through BLAST searches with IFT proteins from *Homo sapiens* as queries, 30 and 27 homologs were detected in *B. natans* and *A. amoebiformis*, respectively (Fig. 3a;  Table S6). We further analyzed the expression levels of these genes (Fig. 3b;  Table S6). TPM values of *A. amoebiformis* IFT genes (0 to 34.3; mean = 6.9) were clearly lower than those of *B. natans* genes (12.3 to 65.6; mean = 32.0). These results suggest that *A. amoebiformis* possesses a core set of flagellar protein genes, however, their expression was suppressed under our culture conditions.

### 3.6. Organelle DNA fragments in the *A. amoebiformis* nuclear genome

*A. amoebiformis* possesses three types of organelle DNAs, mitochondrial DNA (mtDNA), plastid DNA (ptDNA), and nucleomorph DNA (nmDNA). Although these DNAs encode a subset of proteins, many organelle proteins are encoded by the nuclear genome, partly because of EGT. To elucidate whether EGT still occurs in chlorarachniophytes, we performed BLASTN searches to identify organelle-derived sequences in the nuclear genome of *A. amoebiformis*, using its organelle genomes as queries (*e*-value < 1 × 10^−4^, length > 30 bp, sequence identify > 85%). False-positive hits corresponding to non-coding RNA sequences, such as rRNAs, snRNAs, and tRNAs, were manually excluded. We identified four nuclear-integrated ptDNA fragments (NUPTs) of 33, 48, 68, and 339 bp in size, three nuclear-integrated nmDNA fragments (NUNMs) of 60, 61, and 83 bp in size, as well as several nuclear-integrated mtDNA fragments (NUMTs). Sequence identity between the organelle DNA and nuclear genome was 87 to 97% for NUPTs (Fig. S6), and 96 to 100% for NUNMs (Fig. S7). Most NUPTs and NUNMs were located in intron regions of the nuclear genome.

The nuclear genome of *B. natans* contains NUMTs, but lacks both NUNMs and NUPTs,^22^ unlike that of *A. amoebiformis*. This observation supports the limited transfer window hypothesis,^61^ proposing that DNA release via organelle lysis is lethal in cells with a single plastid and nucleomorph, such as *B. natans*. The presence of NUPTs and NUNMs in *A. amoebiformis* possessing multiple plastids and nucleomorphs aligns with this hypothesis. Our findings suggest that DNA migration from all three organelles is ongoing in *A. amoebiformis*, possibly in other chlorarachniophytes with multiple organelles.

### 3.7. Recent mitochondria-to-nucleus gene transfer

It has been reported that two ribosomal protein genes (*rpl16* and *rps4*) were recently transferred from mitochondria to the nucleus of the chlorarachniophyte *Lotharella oceanica*, whereas these two genes are still retained in the mitochondrial genome of *B. natans*.^19^ To further investigate the ongoing EGT events after the divergence of chlorarachniophytes, we examined whether any genes recently disappeared from the organelle genomes of *A. amoebiformis*. Plastid gene content is highly conserved among chlorarachniophyte species, with no species-specific gene loss.^6^ In contrast, the nucleomorph genome of *A. amoebiformis* lacks eight protein-coding genes (*mis3*, *rpl34*, *rpoB*, *rbp11*, *rhel1*, *tcpG*, mcm-like, and ubiquitin-like) that are present in those of other three chlorarachniophytes.^18^ However, counterparts of these missing genes were not identified in the nuclear genome of *A. amoebiformis* by BLASTP searches with an e-value cut-off of 1 × 10^−5^. This suggests that functional gene transfer from the nucleomorph to the nucleus has not occurred since the speciation of this species.

As in the two ribosomal protein genes of *L. oceanica*, two mitochondrial genes, *nad9* (NADH dehydrogenase subunit 9) and *rps11* (ribosomal protein S11), were absent from the mitochondrial genome of *A. amoebiformis* but present in those of *B. natans* and *L. oceanica* (Table S7; Fig. S8). Homologous genes for these two mitochondrial proteins were identified in the nuclear contigs of *A. amoebiformis* and actively expressed (Table S2). Both genes contained spliceosomal introns, further supporting their nuclear origins. Phylogenetic analyses revealed that nuclear-encoded NAD9 and RPS11 were closely related to the mitochondrion-encoded orthologs of other chlorarachniophytes (Fig. S9), strongly suggesting that they were recently acquired through EGT.

**Fig. 4.**
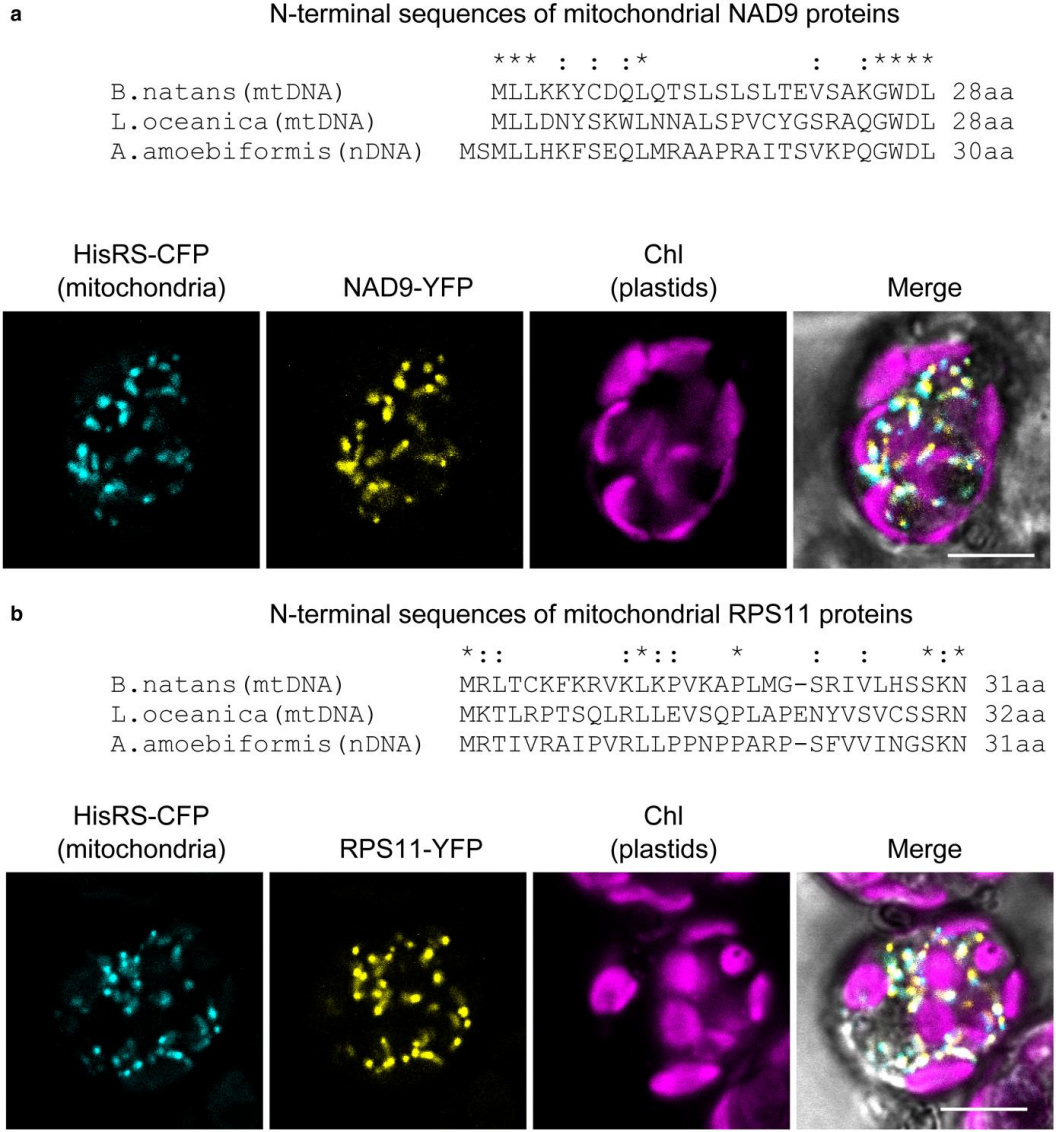
Localization of two nucleus-encoded mitochondrial proteins in *A. amoebiformis*. Alignments show the N-terminal sequences of mitochondrial NAD9 a) and RPS11 b) proteins encoded by mtDNA and nDNA in chlorarachniophytes. Conserved amino acids are marked with asterisks, and shared residues between *A. amoebiformis* and another species are indicated with colons. Confocal images show the subcellular localization of nuclear-encoded NAD9 (a) and RPS11 (b) in *A. amoebiformis*. Mitochondria were labeled by CFP fused to a mitochondria-targeted histidine tRNA synthetase (HisRS-CFP). Signals from YFP-tagged NAD9 and RPS11 overlap with CFP signals in mitochondria. Chl is chlorophyll autofluorescence. Scale bars = 5 µm.

Typically, nuclear-encoded mitochondrial proteins contain an N-terminal presequence that functions as a mitochondrial targeting signal and is cleaved upon import. However, such extensions were not detected in the nuclear-encoded NAD9 and RPS11 of *A. amoebiformis* compared to their mitochondrial counterparts (Fig. 4; Fig. S8). We performed both in silico and in vivo analyses to verify whether these proteins are targeted to the mitochondria. Mitochondrial targeting signals were predicted in mature proteins by at least three of the following programs: TargetP-2.0,^62^ iPSORT,^63^ Predotar,^64^ and PredSL.^65^ Additionally, fluorescent protein-tagging experiments demonstrated that both proteins were localized in the mitochondria (Fig. 4). These results suggest that recent nuclear-transferred mitochondrial proteins lack canonical N-terminal extensions, instead possibly using a part of their mature sequence as a targeting signal. A similar phenomenon has been reported in angiosperms, where mitochondrial *rps10* genes have recently migrated to nuclear genomes in multiple lineages, with some being expressed without acquiring a presequence.^66,67^ Furthermore, some mitochondrion-encoded ribosomal proteins in angiosperms possess an internal latent signal for mitochondrial targeting even before gene transfer.^68^ Therefore, recent mitochondrial gene transfers without gaining an N-terminal presequence are common in eukaryotes.

In this study, we identified recently migrated genes from mtDNA to the nuclear genome of *A. amoebiformis*, but found no evidence of recent EGT from ptDNA or nmDNA, possibly due to the complexity of plastid targeting signals. Mitochondrial targeting signals typically consist of short N-terminal peptides enriched in basic residues. In contrast, nuclear-encoded plastid proteins generally possess an N-terminal bipartite sequence comprising a signal peptide followed by a transit peptide, and are transported via the secretory pathway in chlorarachniophytes.^69,70^ Given this complexity, it is unlikely that a portion of mature proteins would fortuitously function as a plastid targeting signal. Although the presence of NUPTs, NUNMs, and NUMTs indicates ongoing DNA transfer from all three organelles, the requirement to acquire such complex targeting sequences possibly limits the success of EGT from ptDNA and nmDNA in *A. amoebiformis*.

## Supplementary Material

dsaf035_Supplementary_Data

## Data Availability

Raw sequence data of *A. amoebiformis* have been deposited in DDBJ/GenBank/ENA under the accession numbers, DRR687620–DRR687621 (MinION sequencing), DRR796689–DRR796690 (Illumina DNA sequencing), and DRR687622–DRR687625 (Illumina RNA sequencing). Assembly and annotation data of *A. amoebiformis* are available on DDBJ/GenBank/ENA under the accession numbers, BAAHQB010000001–BAAHQB010000567.
