# Supplementary material for: Nuclear genome sequencing reveals the highly intron-rich architecture of the chlorarachniophyte alga Amorphochlora amoebiformis
Source: DNA Res. 2025 Nov 28;32(6):dsaf035. doi: 10.1093/dnares/dsaf035 (PMC12730881; doi:10.1093/dnares/dsaf035)
Supplement: dsaf035_Supplementary_Data [file dsaf035_supplementary_data.zip › Supplemetary figures.pdf]

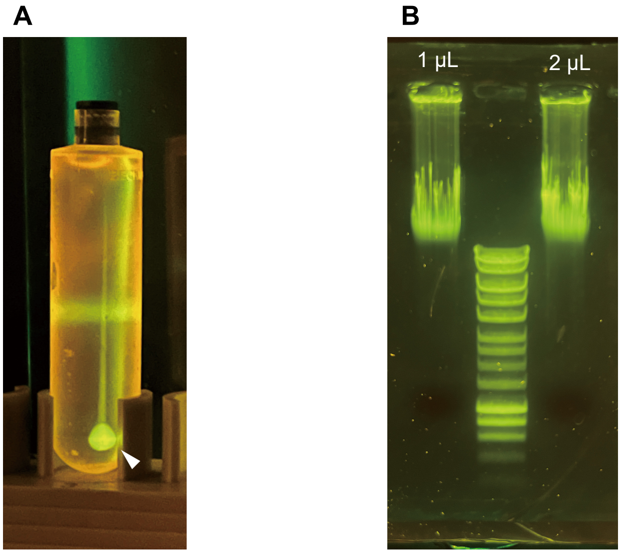

Figure S1. Genomic DNA for sequencing. (A) DNA purification by CsCl density gradient ultracentrifugation. (B) Agarose gel electrophoresis of purified DNA (approximately 1 µg/µL). DNA was visualized under blue LED light with SYBR Safe DNA Gel stain. The molecular weight marker is HyperLadder 1 kb (meridian BIOSCIENCE).

total length: 214195709 bp
GC level: 27.04 %
bases masked: 153836938 bp (71.82 %)

	No.	length	percentage
Retroelements	72608	17534019 bp	8.19 %
DNA transposons	31556	4433715 bp	2.07 %
Unclassified	315824	70651151 bp	32.98 %
Total interspersed repeats:		92618885 bp	43.24 %
Small RNA:	1913	259290 bp	0.12 %
Satellites:	1	225 bp	0.00 %
Simple repeats:	489327	59464426 bp	27.76 %
Low complexity:	9016	957258 bp	0.45 %

total length: 91405885 bp
GC level: 44.85 %
bases masked: 12348109 bp (13.51 %)

	No.	length	percentage
Retroelements	4298	2373834 bp	2.60 %
DNA transposons	1184	87340 bp	0.10 %
Unclassified	34422	3074502 bp	3.36 %
Total interspersed repeats:		5535676 bp	6.06 %
Small RNA:	0	0 bp	0.00 %
Satellites:	0	0 bp	0.00 %
Simple repeats:	167919	6391937 bp	6.99 %
Low complexity:	8780	420496 bp	0.46 %

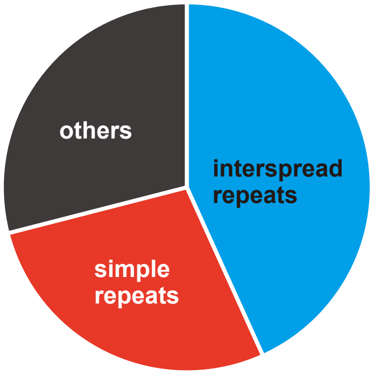

***Amorphochlora* (214 Mbp)**

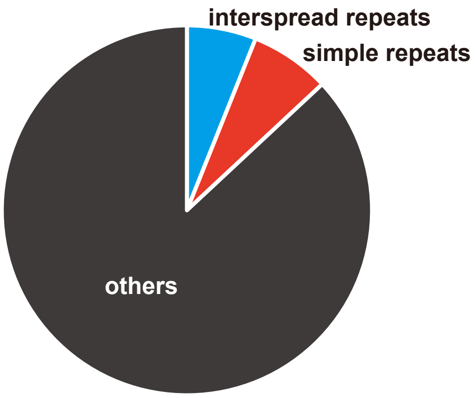

***Bigelowiella* (91 Mbp)**

Figure S2. Repeat elements in the genome sequences of *Amorphochlora amoebiformis* and *Bigelowiella natans* were using RepeatModeler version 2.0.2 and RepeatMasker version 4.1.2 with default settings. The upper tables summarize the results of RepeatMasker and the pie charts represent the percentage of interspread and simple repeats in each genome.

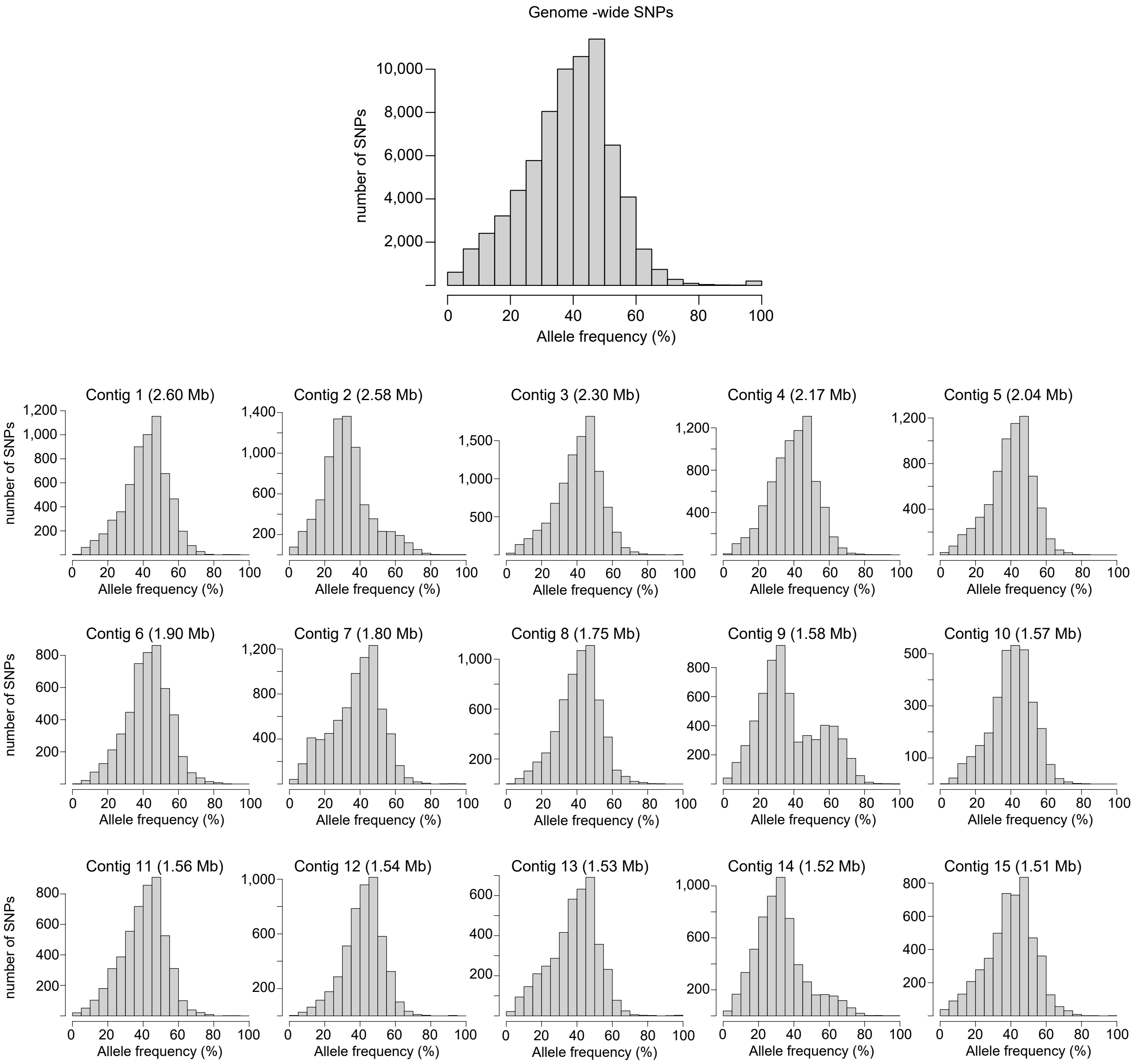

Figure S3. Histograms showing allele frequency distributions for fifteen contigs longer than 1.5 Mb.

The x-axis represents allele frequency, and the y-axis indicates the number of single nucleotide polymorphisms (SNPs).

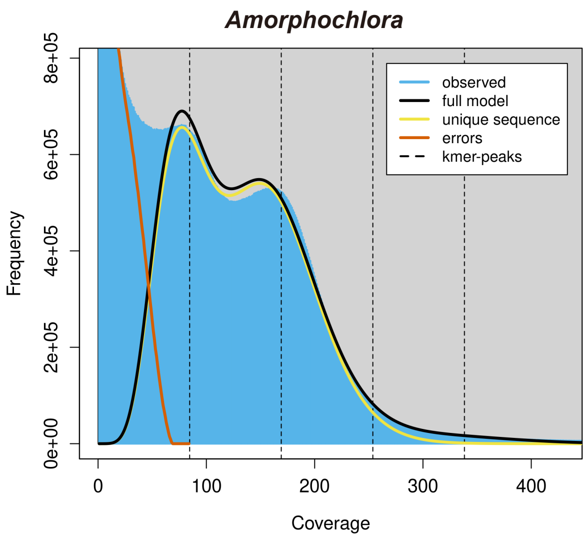

p = 2, k = 21

property	min	max
Homozygous (aa)	98.2233%	98.2821%
Heterozygous (ab)	1.7179%	1.77673%
Model Fit	81.0458%	95.8326%

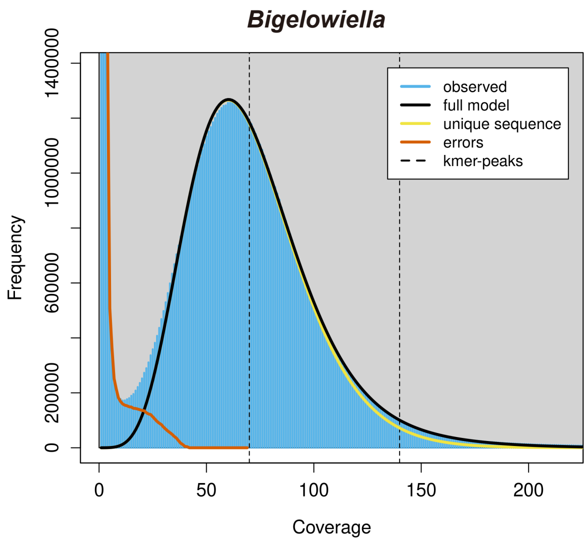

p = 1, k = 21

property	min	max
Homozygous (a)	100%	100%
Model Fit	91.1637%	98.3981%

Figure S4. K-mer frequency distributions of *Amorphochlora amoebiformis* and *Bigelowiella natans.*

Using total 26.1 Gb Illumina short reads (GenBank accession numbers: SRR14100013, DRR796689, and DRR796690) of *A. amoebiformis* and 8.5 Gb Illumina short reads (SRR14100056) of *B. natans*, k-mers were counted using jellyfish v2.3.1 at k = 21.

The Illumina reads were trimmed using fastp v1.0.1. K-mer spectra were visualized using GenomeScope2.

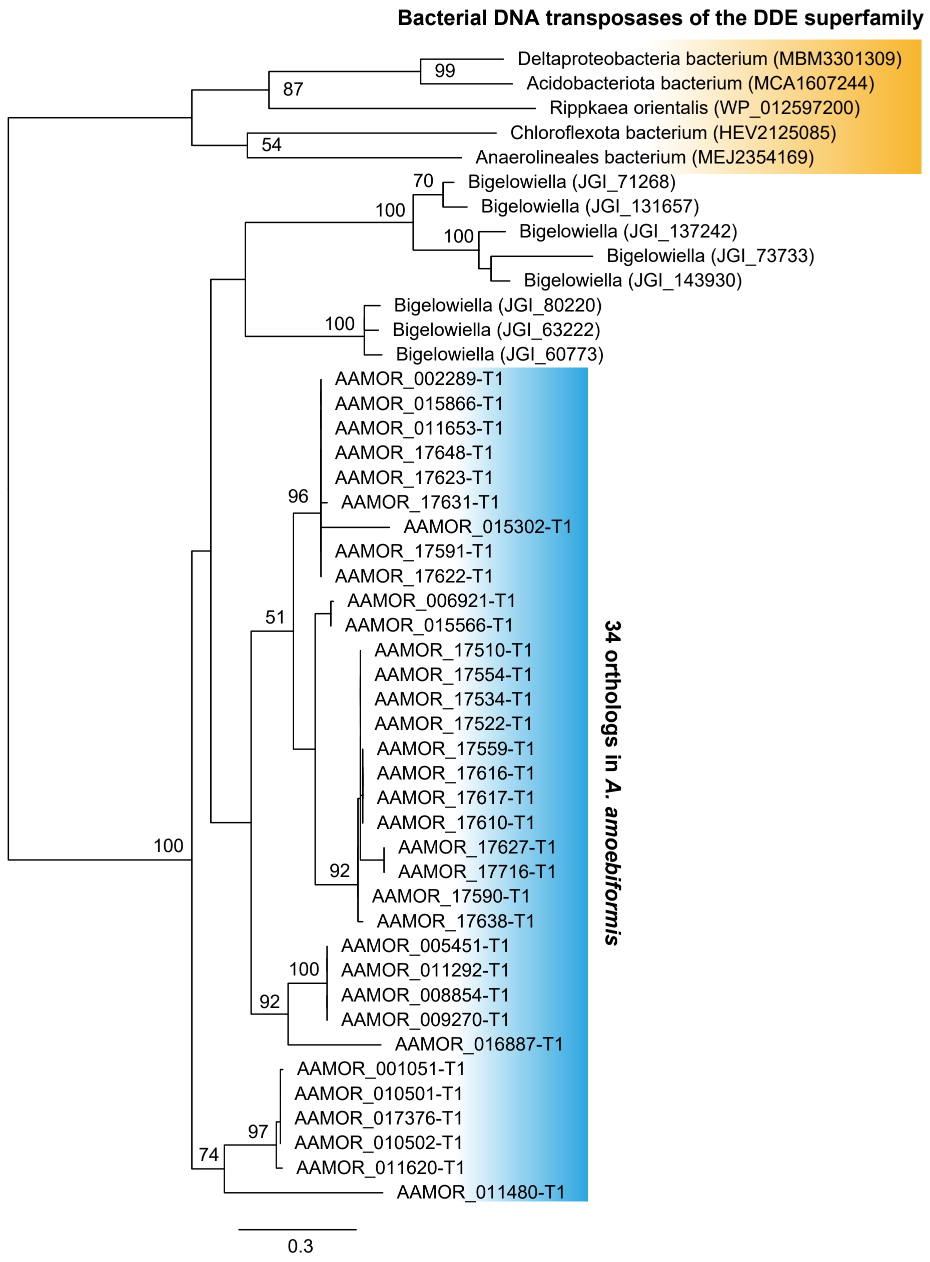

Figure S5. Maximum likelihood tree of DNA transposases belonging to the DDE superfamily.

The tree includes 34 orthologs from *A. amoebiformis* and 8 from *B. natans*. It was constructed using the VT+G4 model implemented in IQ-TREE, based on an alignment of 47 sequences comprising 166 amino acid positions. Bootstrap support values are shown at major nodes (>50%).

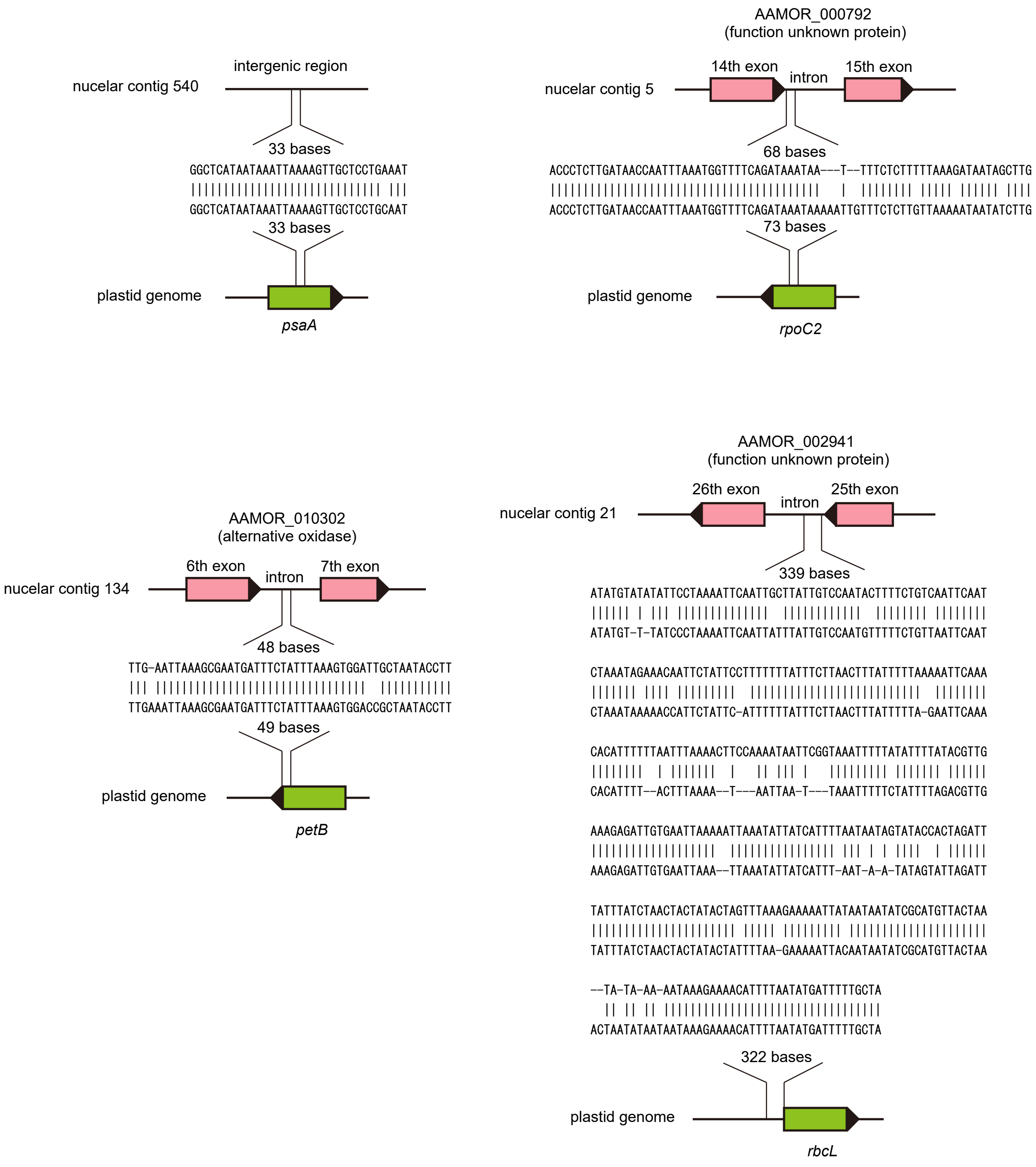

Figure S6. Nuclear sequences of plastid DNA (NUPTs) in *A. amoebiformis*.

Four plastid DNA fragments (33, 48, 68, and 339 bp, respectively) were identified in an intergenic region and introns of different nuclear contigs. Sequence identity is indicated by vertical lines.

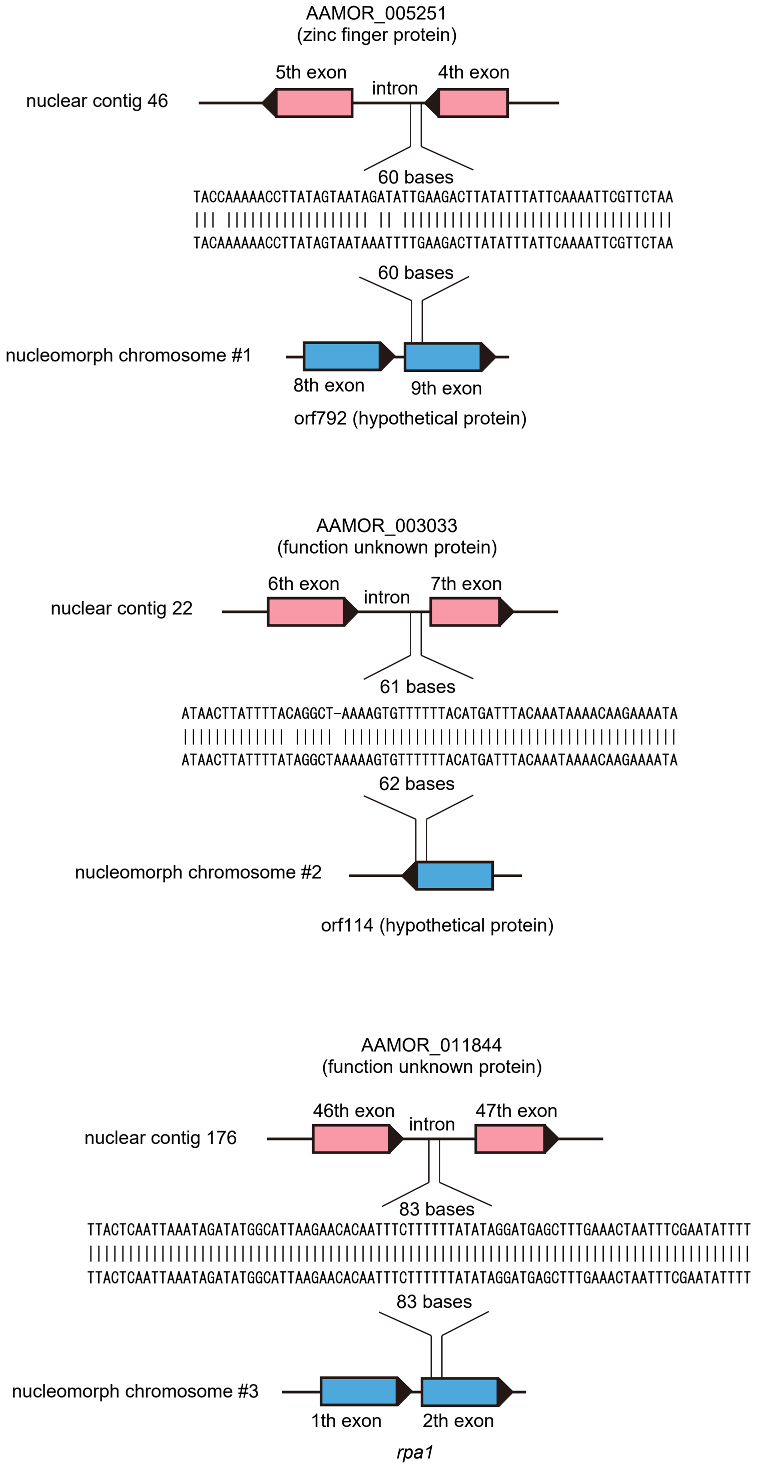

Figure S7. Nuclear sequences of nucleomorph DNA (NUNMs) in *A. amoebiformis*.

Three nucleomorph DNA fragments (60, 61, and 83 bp, respectively) were found in introns of different nuclear contigs. Sequence identity is indicated by vertical line.

A

```
Loce_mtDNA      MKTLRPTSQLRLLEVSQPLAPENYVSVCSSRNNCRISVVRRGRLEGLASVRKSRAYPGYE      60
Bnat_mtDNA      MRLTCKFKRVKLKPVKAPLMG-SRIVLHSSKNTCRMFIVKEGRLVLYTSVKRSSIYTGYE      59
Aamo_nDNA       MRTIVRAIPVRLLPPNPPARP-SFVVINGSKNTTRITCVHEGKVIAHTSTRRSKEYKGTE      59
                *:       ::*     *    : :    *:*  *:  *::*     :* ::*  * * *

Loce_mtDNA      RRLDIASYTFAKDFARRFLRKRERRQASYVLYVKGPKKQAVLGLLDGGMRFLWAIQSFRG      120
Bnat_mtDNA      RRLWIAAYTTALHFGREAQKRKDIRNTKFELYLKGSQKHAVAGFLDSGFVFTKVKEIKNP      119
Aamo_nDNA       RRTAQAIYSVALDFGRELTRSKTIRSSLFDLRIHGLQKNAVLGLLDSGFQFNSIREVKNP      119
                **   * *  * :*:*:  : : :*   : *   * :* **:*:**.*: *    : :::

Loce_mtDNA      PFNGCISAKVRRTGRRKKKHFRRLPRVTGRARSCDLIAIA    160
Bnat_mtDNA      AFNGCFSKKSRRL---------------------------    132
Aamo_nDNA       KFNGCRLRKPRRL---------------------------    132
                 ****   * **
```

B

```
Loce_mtDNA      --MLLDNYSKWLNNALSPVCYGSRAQGWDLVLRVKAEDVVRVLMFLQSSSGSSFKVLSDL      58
Bnat_mtDNA      --MLLKKYCDQLQTSLSLSLTEVSAKGWDLRAHTTASNLVGVLFFIKSSTSSLFNSLSDI      58
Aamo_nDNA       MSMLLHKFSEQLMRAAPRAITSVKPQGWDLVATTTPKDLLPSLFFLKNSSMSQFHGLIDI      60
                  *** : : :*  :     : :  :****:: ::  ::   *:*:: *: * *  * *:

Loce_mtDNA      AVIDRPSSKNRFSLVYNLLSLKYQCRIFLRLTLSEGAGVPSAVPVFAGADWMEREAWDLF      118
Bnat_mtDNA      AASDFPDKAERFELSYNLLSTKYGSRLLLKLTVGEGDVVPSITTLFPGANWMEREAWDLL      118
Aamo_nDNA       VGADYPDRADRFELTYVLLSYQYKTRMLLRTTANETTIVPSAVDVFPSADWHEREVWDMY      120
                   * *:   **:* * ***  *  * :*: *  *   ***:  :*: *:* *** **

Loce_mtDNA      GVFFIGHPDLRRILTDYGFSGHPLRKDFPLTGYFESCYDSGVRRVVYEPVELAQEFRVFS      178
Bnat_mtDNA      GVFFNGHPDLRRILTDYGFEGHPLRKDFPLTGYLEARYDVARARIVYEPVELAQEYRDFS      178
Aamo_nDNA       GIYFGGHPNLRRILTDYGFEGHPLRKDFPLTGYVETRYDATLGRIVYEPVELAQEYRDFS      180
                * :* *** **********:************* * :**    *:**********:*:**

Loce_mtDNA      FPASWK----------------       184
Bnat_mtDNA      FKSPWKT---------------       185
Aamo_nDNA       FNSPWTKIGEELPTAIESGKKE       202
                * ::*
```

Figure S8. Sequence alignments of mitochondrial RPS11 (A) and NAD9 (B) proteins among the three chlorarachniophytes, *Lotharella oceanica*, *Bigelowiella natans*, and *Amorphochlora amoebiformis*. The NAD9 and RPS11 proteins are encoded by the nuclear genome in *A. amoebiformis*, whereas they are mitochondrion-encoded in the other two species. Conserved amino acids are marked with asterisks, and shared residues between *A. amoebiformis* and another species are indicated with colons.

A

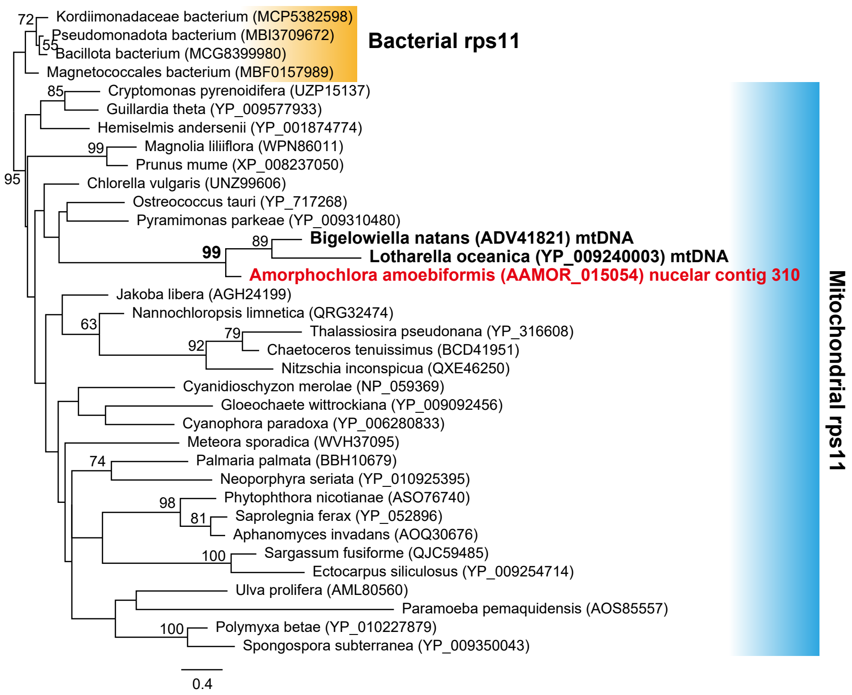

B

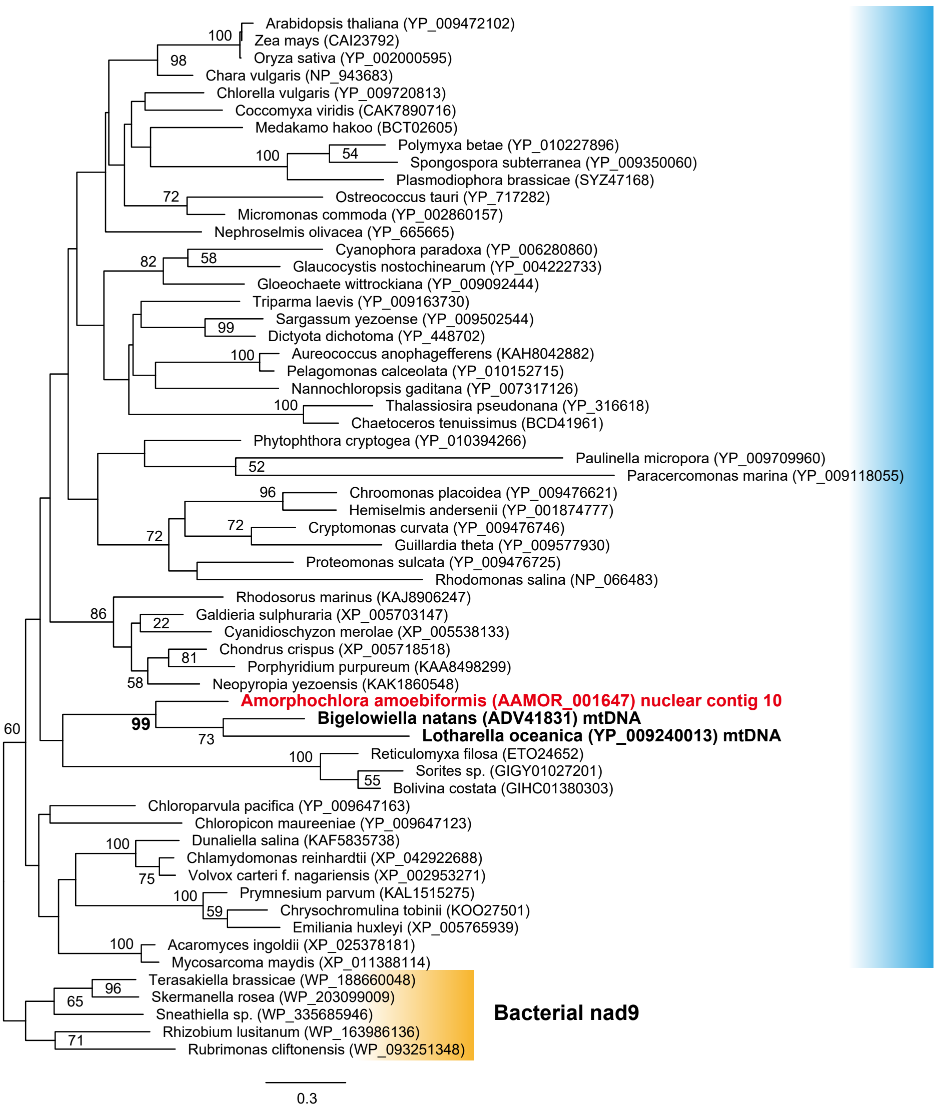

Figure S9. Maximum likelihood trees of mitochondrial RPS11 (A) and NAD9 (B), including several bacterial homologs as outgroup. Trees were constructed using the Q.pfam+F+G4 (RPS11) and the Q.yeast+I+G4 (NAD9) models implemented in IQ-TREE. The RPS11 tree includes 35 sequences with 100 amino acid positions, and the NAD9 tree include 60 sequences with 181 amino acid positions. The values at nodes indicate bootstrap supports when they are higher than 50%.
